# Temperature-dependent iron motion in extremophile rubredoxins – no need for ‘corresponding states’

**DOI:** 10.1038/s41598-024-62261-2

**Published:** 2024-05-28

**Authors:** Francis E. Jenney, Hongxin Wang, Simon J. George, Jin Xiong, Yisong Guo, Leland B. Gee, Juan José Marizcurrena, Susana Castro-Sowinski, Anna Staskiewicz, Yoshitaka Yoda, Michael Y. Hu, Kenji Tamasaku, Nobumoto Nagasawa, Lei Li, Hiroaki Matsuura, Tzanko Doukov, Stephen P. Cramer

**Affiliations:** 1 Georgia Campus, Philadelphia College of Osteopathic Medicine, Suwanee, GA 30024 USA; 2 SETI Institute, Mountain View, CA 94043 USA; 3 Department of Chemistry, Carnegie Mellon University, Pittsburgh, PA 15213 USA; 4 LCLS, SLAC National Laboratory, Stanford, CA 94025 USA; 5 Universidad de La República, Montevideo, Uruguay; 6 Precision Spectroscopy Division, SPring-8/JASRI, Sayo, Hyogo 679-5198 Japan; 7 Advanced Photon Source, Argonne National Laboratory, Lemont, IL 60439 USA; 8 RIKEN/SPring-8 Center, Hyogo, 679-5148 Japan; 9 Synchrotron Radiation Research Center, Hyogo, 679-5165 Japan; 10 SSRL, SLAC National Laboratory, Stanford, CA 94025 USA

**Keywords:** Rubredoxin, Iron-Sulfur, Extremophile, Hyperthermophile, Psychrophile, Corresponding States, Bioinorganic chemistry, Enzyme mechanisms, Metals, Proteins, Computational biophysics, Molecular biophysics

## Abstract

Extremophile organisms are known that can metabolize at temperatures down to − 25 °C (psychrophiles) and up to 122 °C (hyperthermophiles). Understanding viability under extreme conditions is relevant for human health, biotechnological applications, and our search for life elsewhere in the universe. Information about the stability and dynamics of proteins under environmental extremes is an important factor in this regard. Here we compare the dynamics of small Fe-S proteins – rubredoxins – from psychrophilic and hyperthermophilic microorganisms, using three different nuclear techniques as well as molecular dynamics calculations to quantify motion at the Fe site. The theory of ‘corresponding states’ posits that homologous proteins from different extremophiles have comparable flexibilities at the optimum growth temperatures of their respective organisms. Although ‘corresponding states’ would predict greater flexibility for rubredoxins that operate at low temperatures, we find that from 4 to 300 K, the dynamics of the Fe sites in these homologous proteins are essentially equivalent.

## Introduction

Over the past 4 decades, our knowledge about life under extreme conditions has dramatically expanded^1,2^. Organisms have been found that can function down to -25 °C (psychrophiles) and up to 122 °C (hyperthermophiles)^3^. Microbial activity has been demonstrated at GPa pressures^4^, and growth at 100 MPa can has also been demonstrated (piezophiles)^5,6^, as well as over a wide range of pH values (acidophiles and alkaliphiles)^7^, ionic strengths (halophiles)^8^, and other stressors and even combinations thereof^9^. These living systems must modulate their protein properties to function best in their preferred environment. Understanding how these extremophiles do so is interesting in its own right. It is also relevant for human health, biotechnological applications, and our search for life elsewhere in the universe^10^.

The dynamics of proteins in their proteins is an important part of extremophile adaptation. The temperature-dependent flexibility of enzymes is relevant to the optimum growth temperature for an organism and the range over which it can survive. Here the flexibility is defined as the rms deviation of atomic positions: $\sqrt{\langle {\mu }^{2}\rangle }$. According to the ‘corresponding states’ hypothesis, enzymes have evolved to have comparable flexibility at the ideal growth temperature for their respective organism^11,12^. In a similar vein, it is often stated that “directed thermal motion is needed for catalysis” and that “flexibility is necessary to allow catalysis at a metabolically appropriate rate”^13,14^. In support of these ideas, neutron scattering experiments found comparable flexibility for psychrophiles and thermophiles at their respective adaptation temperatures (from 4 to 85 °C)^15^.

However, the ‘corresponding states’ model is not universally accepted, and some experiments conflict with its predictions. For hyperthermophilic *Pyrococcus furiosus* rubredoxin (*Pf* Rd), NMR-monitored amide hydrogen exchange experiments found larger flexibility comparable to the mesophile *Clostridium pasteurianum* (*Cp*) protein^16,17^. Using molecular dynamics calculations, Grottesi and coworkers found that at 300 or 373 K, hyperthermophilic *Pf* Rd is more flexible than the homologous mesophilic Rd from *Clostridium pasteurianum*^18^. Yet, a decade later, Rader compared the same pair with MD and explained the greater thermostability of *Pf* Rd *vs*. *Cp* Rd as the result of a ‘decrease in flexibility’^19^. In a similar vein, from neutron scattering, NMR, and other measurements, hyperthermophilic P450 CYP119 was found to be more flexible than its mesophilic counterpart CYP101A at all temperatures above 200 K^20^. In view of the discordance between the ‘corresponding states’ theory and multiple experiments, we decided to investigate the flexibility of different Rds at the Fe site by a variety of methods.

**Figure 1:**
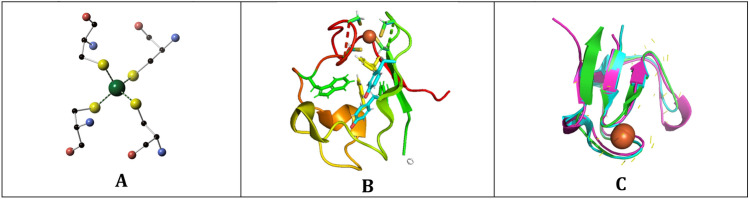
(**A**) Ball and stick picture of the Fe(S-Cys)_4_ site in *Pf* Rd. (**B**) Pymol diagram accentuating aromatic amino acids in *Pf* Rd. (**C**) Ribbon diagrams of *Pf* Rd with homology structure of *Px* Rd.

Rubredoxins (Rds) are the simplest Fe-S proteins^21^; they have an Fe(S-Cys)_4_ center and molecular masses on the order of 6 kDa (Fig. 1). Rubredoxins are electron transfer proteins^22^, and they play important roles in photosystem II assembly^23^, as redox partners of alkane hydroxylases^24,25^ and P-450 enzymes^26^, and as platforms for artificial catalysts^27,28^. As such they are an ideal test system for studies of extremophile dynamics. The structures and dynamical properties of rubredoxins have been studied by a variety of techniques and over ~ 10 orders of magnitude time scales. The experimental methods vary from inelastic neutron scattering on the picosecond scale^16,29^ to amide H–D exchange experiments over tens of seconds^30,31^. Rubredoxins have also been extensively investigated by molecular dynamics (MD)^18,32–37^.

For this study we have compared Rds from the hyperthermophile *Pyrococcus furiosus* (*Pf*)^38^ with those from the psychrotolerant organisms *Pseudomonas* sp. strain AU10 (*Px*)^39^ and *Polaromonas glacialis* (*Pg*). *Pf* is a marine archaeon with an optimal growth temperature of ~ 100 °C^40^. *Px* is an Antarctic freshwater organism with an optimal growth temperature of 28 °C that can still grow close to 0 °C^39^. *Pg* has been isolated from alpine glaciers and grows well from 1 to 25 °C^41,42^. For *Pf*, it is presumed that *Pf* Rd is reduced by NADPH rubredoxin oxidoreductase (NROR), and that *Pf* Rd in turn reduces either a superoxide reductase (SOR) or a peroxide-reducing rubrerythrin (Rr)^43^. For the psychrophilic Rds, since these species are implicated in the biodegradation of hydrocarbons and xenobiotics, one can assume that *Pg* and *Px* Rd provide electrons to enzymes that metabolize such molecules.

We have quantified the amount of ^57^Fe motion in these rubredoxins by measuring the Lamb-Mössbauer factor as a function of temperature. The Lamb-Mössbauer factor, *f*_LM_, is the ratio of elastic line intensity to overall nuclear absorption. In the case of harmonic motion ${f}_{LM}=\text{exp}(-{k}^{2}\langle {\mu }^{2}\rangle )$, where *k* = 2π/λ and $\langle {\mu }^{2}\rangle$ is the mean square motion of the resonant nucleus. This breaks down when the motion is anharmonic, but ${f}_{LM}$ remains a useful metric.

We have derived ${f}_{LM}$ from three different measurements as well as from molecular dynamics calculations. One approach has been Nuclear Resonance Vibrational Spectroscopy (NRVS), where the elastic and inelastic intensities can be directly compared^44–47^. We also conducted conventional Mössbauer experiments on these Rds. In this case we obtained relative ${f}_{LM}$ values over a range of temperatures and then calibrated an absolute value at the low temperature limit with respect to the NRVS results. A third approach, also using synchrotron radiation, obtains relative ${f}_{LM}$ values from the intensity of the Nuclear Forward Scattering (NFS)^48,49^. Finally, the values from these three experimental methods have been compared with each other and with molecular dynamics calculations. In preliminary feasibility studies, we found that the oxidized Fe(III) form of rubredoxin was susceptible to photoreduction at higher temperatures. Thus, for all of these initial dynamics studies, we have focused on the reduced Fe(II) form of rubredoxin, which did not show any spectral changes during data collection.

The original Mössbauer approach to protein dynamics^50^ and its interpretation as a protein dynamical transition have been criticized^51,52^. NRVS and NFS are alternative and complementary probes, and ${f}_{LM}$ can be reliably extracted from both NRVS and NFS data^53^. *All three of these methods sense motion of a single isotopic type of nucleus, in this case Fe-57, compared to neutron scattering which averages over (mostly protons) for an entire protein or organism.*

## Experimental results

### Nuclear resonance vibrational spectroscopy (NRVS)

**Figure 2:**
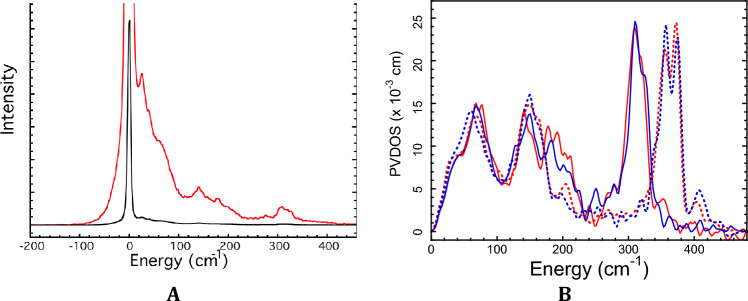
(**A**) Raw NRVS at 37K for *Pf* Rd before subtraction of elastic peak and normalization (Red line), scaled so that vibrational structure is visible. The (Black line) curve is the 37K data reduced by a factor of 100. (**B**) Processed NRVS data after removal of the elastic peak and normalization to PVDOS: reduced *Pf* Rd 46K (Red line), reduced *Pg* Rd 48K (Blue line), oxidized *Pf* Rd 46K (Red dotted line), oxidized *Pg* Rd 52K (Blue dotted line).

Since the procedure for deriving Lamb-Mössbauer factors from NRVS is not that common in bioinorganic chemistry, we first illustrate the temperature-dependent ^57^Fe PVDOS (Partial Vibrational Density Of States) and normalization procedures in Fig. 2. The data processing involves estimation and removal of the elastic peak due to conventional Mössbauer scattering and a Fourier transform procedure that removes multiphonon events and yields a normalized PVDOS^54^.

**Figure 3:**
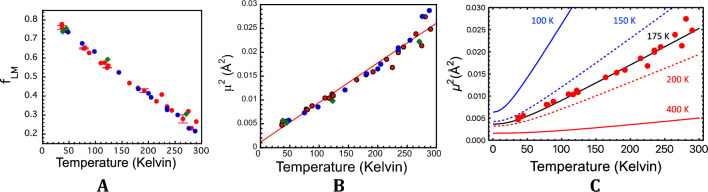
(**A**) NRVS-derived Lamb-Mössbauer factors *vs*. T for ^57^Fe in *Pf* Rd (Red filled circle), *Pg* Rd (Blue filled circle), and *Px* Rd (Green filled diamond). (**B**) Comparison of NRVS-derived < *u*^2^ > for the ^57^Fe sites in *Pf* Rd (Red filled circle), *Pg* Rd (Blue filled circle), and *Px* Rd (Green filled diamond). Straight line is linear fit to just *Pf* Rd data. (**C**) < *u*^2^ > *vs*. temperature for different Debye temperatures as labeled, compared with experimental values from *Pf* Rd NRVS (Red filled circle).

In Fig. 3A,B, we compare NRVS-derived values for the Lamb-Mössbauer factors and < *u*^2^ > for the ^57^Fe sites in all three rubredoxins over a range of temperatures. The trend for *Pf* Rd is smooth and approximately linear over most of the temperature range. There is no evidence for a so-called ‘dynamical transition’ involving a rapid change in slope in the region above 200 K, as derived from conventional Mössbauer spectroscopy for myoglobin^53,55,56^. Finally, within experimental error, at comparable temperatures, the < *u*^2^ > is the same for the psychrotolerant *Px* Rd, the psychrophilic *Pg* Rd, and the hyperthermophilic *Pf* Rd.

In Fig. 3C, the NRVS-derived < *u*^2^ > for *Pf* Rd is compared with theoretical curves for Debye models^57^ with a variety of temperatures. There is good agreement with a Debye temperature of ~ 175 K. Previous work by Debrunner and coworkers put the Rd Debye temperature between 145 and 212 K^58^. Using 175 K, the Debye model predicts < *u*^2^ >  = 0.00375 Å^2^ at 4.2 K and hence a low-temperature Lamb-Mössbauer factor ${f}_{LM}=$ 0.82. This compares favorably with the Lamb-Mössbauer factor ${f}_{LM}=$ 0.85 ± 0.06 calculated for *Cp* rubredoxin from 4.2 K Mössbauer data by Debrunner and coworkers^58^. The temperature dependence of their data was also consistent with our results.

The calculated mean square Fe motion in Fig. 3B is predicated on the assumption of harmonic motion for the ^57^Fe nucleus, and anharmonic effects can require corrections^59–62^.

### Temperature-dependent Rubredoxin Mössbauer

**Figure 4:**
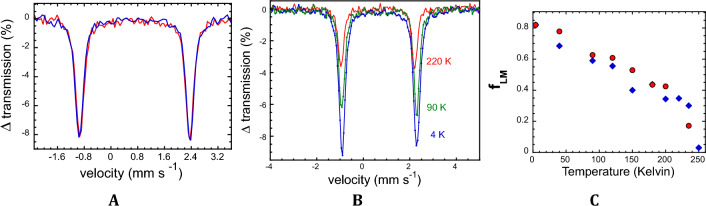
(**A**) Low temperature (4.2 K) Mössbauer spectra for reduced *Pf* (Red line) and *Pg* (Blue line) Rd. The *Pg* Rd intensity was scaled to match *Pf* Rd, but the velocity scales were unchanged. (**B**) Representative Mössbauer spectra for *Pf* Rd at different temperatures. (**C**) Lamb-Mössbauer factors *f*_LM_ for *Pf* (Red filled circle) and *Pg* (Blue filled circle) Rd *vs*. temperature, using *f*_LM_ normalized to 0.82 at 4.2 K. *Pf* and *Pg* data superimpose at 4 K and 180 K.

One difference between the NRVS-derived < *u*^2^ > and the previous Mössbauer results for Rd^58^ or heme proteins^50,63^ is the time scale of the experiments. NRVS reflects a time scale on the order of 4 picoseconds, while the lifetime of a Mössbauer measurement is on the order of 100 ns^63^. Accordingly, we also ran the conventional Mössbauer for reduced *Pf* Rd and *Pg* Rd, and the results are presented in Fig. 4. At 4 K the Rd spectra are virtually identical (Fig. 4A) with respective quadrupole splittings of 3.28–3.31 mm s^−1^ and equal isomer shifts of 0.67 mm s^−1^. With increasing temperature, the absorption as governed by *f*_LM_ decreases dramatically (Fig. 4B,C). It has often been noted, that, “*precise measurement of f*_*LM*_* is difficult with conventional Mossbauer spectroscopy*^48^”, and “*it is difficult to determine absolute values for the recoilless fractions … without tedious and difficult experimentation*^64^.” So, the scatter in the observed values is not unexpected.

The Mössbauer data for both rubredoxins shows a dramatic falloff around 235 K. In this regard it is consistent with previous work on Mb that posited a dynamical transition above 200 K^55,56,63^. Thus, as with the previous Mb work, we are seeing significantly different mean square Fe motion on the time scales of the Mössbauer and NRVS measurements. We note that the time-scale of the Mössbauer measurement is governed by the ~ 100 ns excited state lifetime. In contrast, it has been argued that the NRVS time scale is on the order of 4 ps^63^.

To obtain an absolute value for the Lamb-Mössbauer factor, we used the low temperature (4.2 K) limit of the Debye model with a Debye temperature of 175 Kelvin (Fig. 3C). This gave μ^2^ = 3.66 × 10^–3^ Å^2^ and hence ${f}_{LM}=$ 0.82. This value is reasonably close to the 4.9 × 10^–3^ Å^2^ value for < *u*^2^ > derived from the lowest temperature NRVS data at 37 K. We thus feel justified in using ${f}_{LM}=$ 0.82 as the low temperature limit with which to calibrate the 4 K Mössbauer data. Our 0.82 value is similar to the ${f}_{LM}=$ 0.85 ± 0.06 reported by Debrunner et al. for oxidized *Pf* Rd as far back as 1979^58^. This allows us to report absolute ${f}_{LM}$ in Table 1.

**Table 1:** Mössbauer and NFS Results on Reduced Fe(II) *Pf* and *Pg* Rd ^∞^

T (Kelvin)	Linewidth (mm -s^−1^)		ΔE_Q_ (mm s^−1^)				Lamb-Mössbauer Factor*			
			*Pf* Rd		*Pg* Rd		*Pf* Rd		*Pg* Rd	
	*Pf* Rd	*Pg* Rd	Möss	NFS	Möss	NFS	Möss	NFS	Möss	NFS
4.2	0.28	0.28	3.28		3.31		0.82		0.82	
18				3.24		3.27		0.82		0.84
40	0.28	0.27	3.28	3.24	3.32	3.27	0.78	0.77	0.69	0.78
90	0.28	0.27	3.26	3.23	3.30	3.26	0.63	0.65	0.59	0.66
100				3.22		3.26	0.62	0.63	0.58	0.63
120	0.27	0.26	3.25	3.21	3.28	3.25	0.60	0.58	0.55	0.58
150	0.27	0.26	3.24	3.20	3.27	3.24	0.59	0.51	0.40	0.51
180	0.27	0.26	3.23	3.19	3.27	3.23	0.53	0.43	0.38	0.43
200	0.27	0.26	3.22	3.18	3.26	3.22	0.44	0.39	0.50	0.38
220	0.26	0.26	3.21	3.17	3.26	3.21	0.42	0.34	0.32	0.33
235	0.40	0.40	3.20	3.17	3.24	3.20	0.17	0.25	0.34	0.25
250								0.04	≤ 0.05	0.04

*Assuming μ^2^ = 3.6 × 10^−3^ Å^2^ at 4 K.

^∞^Interpolated as necessary.

### Temperature-dependent Rubredoxin nuclear forward scattering

Nuclear forward scattering is a synchrotron technique that provides Mössbauer-like information from the time-dependent scattering of the emitted radiation. Interference between the different scattered frequencies produced by quadrupole and/or magnetic interactions generates ‘quantum beats’ in the scattered intensity, and there can also be ‘dynamical beats’ in the scattered intensity due to absorption and reemission of the beam as it passes through the sample^65^. As early as 1994, Bergmann and coworkers used NFS to extract temperature dependent *f*_*LM*_ for Fe metal^48^.

The properties of the scattered radiation depend on the effective sample thickness χ, where χ =  η_s_ *d *σ_0_${f}_{LM}.$ Here, η_s_ is the volume density of elastic scatterers, *d* is the sample thickness, and σ_0_ is the maximum resonance cross section^65^. In the case of a quadrupole split Mössbauer spectrum, there are two scattered frequencies with splitting $\Delta \omega$, and the transmitted intensity *I*_*tr*_(*t*) can be described as a function of time *t* by^65^:

1 $${{\varvec{I}}}_{{\varvec{t}}{\varvec{r}}}\left({\varvec{t}}\right)\propto \mathbf{exp}\left(-{\varvec{\tau}}\right)\boldsymbol{ }\frac{{\varvec{\chi}}}{{\varvec{\tau}}}\boldsymbol{ }{{\varvec{J}}}_{1}^{2}\left(\sqrt{0.5\boldsymbol{ }{\varvec{\chi}}\boldsymbol{ }{\varvec{\tau}}}\right)\boldsymbol{ }{{\varvec{c}}{\varvec{o}}{\varvec{s}}}^{2}\left(\frac{{\varvec{\Delta}}{\varvec{\omega}}}{2}{\varvec{t}}+\boldsymbol{ }\frac{{\varvec{\chi}}\boldsymbol{ }{{\varvec{\Gamma}}}_{0}}{8\boldsymbol{ }{\varvec{\Delta}}{\varvec{E}}}\right)$$

In this equation, τ is the time in units of the nuclear lifetime τ_0_ and J_1_ is a Bessel function of the first kind. The quadrupole splitting leads to a beat time^65^:

2 $${{\varvec{T}}}_{{\varvec{q}}{\varvec{b}}}=\boldsymbol{ }\frac{2{\varvec{\pi}}}{{\varvec{\Delta}}{\varvec{\omega}}}=\boldsymbol{ }\frac{86\boldsymbol{ }{\varvec{n}}{\varvec{s}}}{{\varvec{\Delta}}{{\varvec{E}}}_{{\varvec{Q}}}[{\varvec{m}}{\varvec{m}}\boldsymbol{ }{{\varvec{s}}}^{-1}]}$$

Hence, the quadrupole splitting can be obtained from the beats: ΔE_Q_ [mm s^−1^] = 86 ns/*T*_qb_.

**Figure 5:**
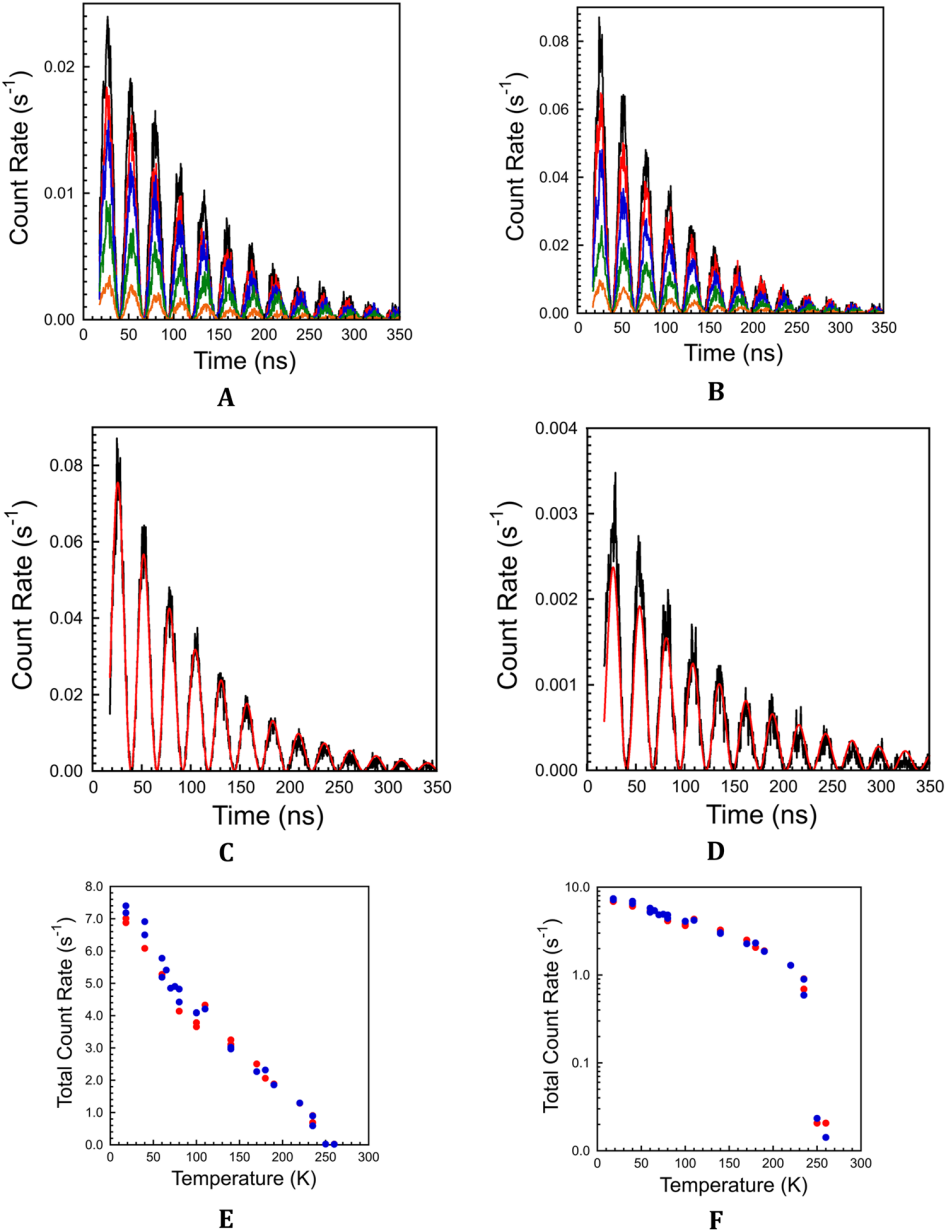
Nuclear forward scattering data. (**A**) *Pg* Rd (in order of decreasing intensity) measured at 18 K (Black line), 60 K (Red line), 110 K (Blue line), 170 K (Green line), and 235 K (Yellow line). Max overall count rate was ~ 1.4 × 10^7^ s^−1^. Bin-width = 0.4675 ns. (**B**) Same for *Pf* Rd. (**C**) Least squares fit (Red line) and data (Black line) for *Pg* Rd NFS at 18 K. (**D**) Least squares fit (Red line) and data (Black line) for *Pf* Rd NFS at 235 K. (**E**) Normalized count rate for *Pf* (Red filled circle) *vs*. *Pg* (Blue filled circle) Rd. (**F**) Same on log scale.

The NFS signals for both Rds are compared in Fig. 5A,B. A least-squares fit of the *Pg* Rd NFS data at 18 K to a version of Eq. (1) is shown in Fig. 5C. The optimized value Δω = 0.240 yields a ΔE_Q_ = 3.27 mm s^−1^, compared with the low temperature Mössbauer value of 3.31 s^-1^. At the other temperature extreme, fitting the *Pf* Rd NFS data at 235 K yields ΔE_Q_ = 3.17 mm s^−1^ (Fig. 5D), compared to the Mössbauer value of 3.20 mm s^−1^.

As seen in Eq. (1), the dependence of the NFS signal amplitude on *f*_LM_ is more complicated. As described in Supporting Information, the bottom line is that for concentrated samples over long times, the NFS signal under these conditions becomes approximately proportional to the Lamb-Mössbauer factor. However, for relatively thin samples, χ ≤ 1, and at short times, the NFS signal will be approximately proportional to χ^2^ and hence to the square of the Lamb-Mössbauer factor: I(t)∼χ^2^exp[-(1 +  χ)τ]^66^.

The temperature dependence of the NFS signals for both Rds is compared in Table 1, Fig. 5A,B,E,F. We see that there is a precipitous drop in the signal around 235 K, just as observed in the Mössbauer data. In other work the same effect has been seen in the NFS of myoglobin^67^, and it has been taken as evidence for a ‘dynamical transition’^63^ or ‘glass transition’^51,68^. Of note is that this transition is not observed in our NRVS data. The agreement between NFS and Mössbauer measurements likely reflects that they explore the same ~ 100 ns time scale, compared to the much shorter time scale for NRVS.

Apart from *f*_LM_, the NFS reveals the temperature dependence of the quadrupole splitting ΔE. Here we see the same trend as in the Mössbauer, but our values tend to be ~ 1% smaller than obtained by the conventional technique.

### Molecular dynamics calculations for T-dependent ^57^Fe motion

**Figure 6:**
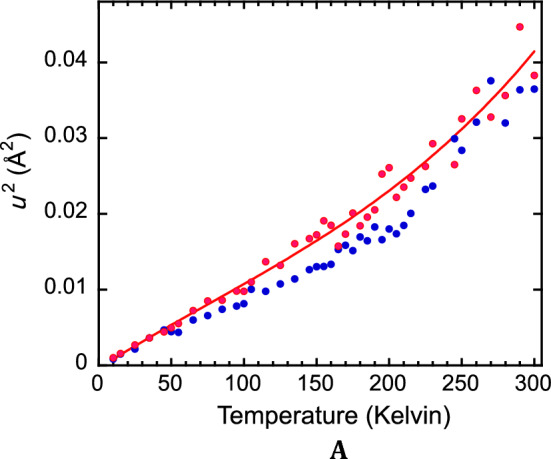
Molecular dynamics predictions for ^57^Fe motion as a function of temperature. Time scales for these calculations were 4 ns. (**A**) Average of 3 MD runs for *Pf* Rd (Red filled circle) *vs.* 1 run for *Pg* Rd (Blue filled circle). Smooth curve is quadratic fit to *Pf* Rd calculations to guide the eye and allow interpolation for Table 2.

The amount of motion at the ^57^Fe site can also be derived from molecular dynamics calculations. As computer technology has improved, such studies of rubredoxin have examined dynamics on progressively longer time scales: from 10 ps in 1993^32^, to 30 ps^35^, 100 ps^34^ 400 ps^36^ 6 ns^18^ and recently out to 1 μs Sala^37^. Our current results on the 4 ns time scale are illustrated in Fig. 6.

**Table 2:** Reduced Fe(II) *Pf* Rubredoxin T-Dependent ^57^Fe RMS Disorder (Å) by Various Techniques.

T T (Kelvin)	Diffraction	NRVS	Mössbauer	NFS	molecular dynamics^§^
4	–	0.06^‡^	0.06	–	–
40	–	0.075	0.068	0.067	0.068
100	0.17^¶**x**^ 0.20^**† x**^	0.10	0.074	0.093	0.10
110	0.23^**& x**^	0.10	0.10		0.11
123	0.28^**Ω x**^	0.11	0.10		0.11
160	0.30^**z x**^	0.12	0.12	0.12	0.13
200	0.27^**z x**^	0.13	0.13	0.14	0.15
235	0.34^**z x**^	0.14	0.18	0.16	0.17
250		0.15	–	–	0.18
295	0.33^**∞ x**^ 0.34^**§ n**^	0.16	–	–	0.20

^x^x-ray diffraction. ^n^neutron diffraction ^z^unpublished *Pf* Rd ^‡^normalized to Debye curve.

^†^PDB 5OME ^∞^PDB 4AR6. ^§^PDB 4AR4 ^‡^ **PDB 2DSX PDB 3KYU ^**Ω**^ 1BRF ^**&**^2DSX (*Dg* Rd).

^‡^Extrapolated from Debye model.

^§^Interpolated from smooth curve fitted to average of 3 simulations.

Over the entire temperature range, from cryogenic temperatures to 300 K, there is relatively little difference in the calculated Fe motion between both proteins. This is in agreement with the experimental results from the previous nuclear spectroscopies. The amount of motion is slightly higher than obtained from NRVS, this may reflect the 4 ns timescale for the calculations *vs.* the ps timescale for NRVS. A brief look at 4 ps time-scale calculations found ^57^Fe rmsd were reduced by ~ 20%, bringing them more in line with NRVS.

## Summary of different approaches

The values for root mean square ^57^Fe disorder derived from different techniques are summarized in Table 2. At 235 K, supposedly above the PDT temperature, there is excellent agreement between NRVS, Mössbauer, and MD techniques for reduced *Pf* Rd ^57^Fe RMSD: 0.16 ± 0.01 Å. The agreement is reasonably good at lower temperatures. However, above 235 K, the signals from Mössbauer and NFS fall sharply, whereas the NRVS is still measurable.

In comparison, the RMSD values obtained via B-values from x-ray and neutron diffraction structures are much larger than the spectroscopic or MD determinations. For example, in the region near 100 K, all of the spectroscopic rmsd estimates are at or below 0.1 Å, whereas the smallest crystallographic values are larger: 0.20 Å for reduced *Pf* Rd at 100 K^69^ and 0.17 Å for oxidized *Pf* Rd at 100 K^69^. The pattern repeats at 295 K, where the diffraction values (0.33 Å or 0.34 Å) are again about twice the estimates from NRVS (0.17 Å) or MD calculations (0.20 Å). The larger diffraction values arise because these methods are sensitive to both static and dynamic disorder in the Fe positions.

## Discussion

In this work we have compared two psychrophilic rubredoxins, *Pg* Rd and *Px* Rd, with a hyperthermophilic homologue from *Pf* Rd. In contrast with previous work which has primarily focused on the dynamics of protein protons, we have used nuclear spectroscopies that solely measure the dynamics of the labeled ^57^Fe site. According to the ‘corresponding states’ hypothesis, the hyperthermophilic Rd should be less flexible than the psychrophilic protein at the same temperature.

Instead, we find that over a wide temperature range, from cryogenic temperatures up to ambient conditions, the dynamics at the Fe redox site are quite similar for both psychrophilic and hyperthermophilic proteins. Our results are thus consistent with hydrogen exchange studies by Hernández et al*.* that found no evidence “systematic rigidification” in *Pf* Rd^16^. In their MD simulations, Grottesi et al. even found greater flexibility for *Pf* Rd compared to mesophilic *Clostridium pasteurianum* (*Cp*) Rd^18^. In another HD exchange study comparing *Pf* and *Cp* Rds, LeMaster et al*.* found “no necessary correlation between thermostabilization and increased conformational rigidity”^70^. These results along with our findings differ from the MD calculations of Rader who found a decreased flexibility for *Pf* Rd compared to *Cp* Rd^71^.

Of course, our use of nuclear probes that focus on motion at the Fe site raises question about the broader implications for protein dynamics of the whole protein. Although the Fe is situated at one end of the molecule, in previous work we have shown that Fe participates in low frequency modes that “*involve concerted in-phase collective motion of large segments of polypeptide*^72^^,^^73^.” Others have proposed that even the Fe–S modes are *“extensively coupled to deformations of the polypeptide backbone*^74^^,^^75^*.”* Thus, although our probes were Fe-centered, rubredoxins are such small proteins that Fe motion should be a good proxy for global protein dynamics.

Our results raise the question: how important is flexibility for the electron transfer role of rubredoxin? Although there are as yet no structures for our Rds bound to redox partners, we can learn from the *Pseudomonas aeruginosa* system. The *Pa* rubredoxin, Rdx, receives electrons from an FADH-based reductase, RdxR, and transfers them to alkane hydroxylases AlkB1 and AlkB2^24^. The structure of a complex between Rd and RdxR has been solved to 2.45 Å by x-ray crystallography^76^. Using an AlfaFold model for the isolated Rdx, we estimate that the average structural change in the Rdx molecule before and after binding RdxR is on the order of 0.5 Å^76^. The flexibility associated with typical enzymatic conformational changes does not seem required for Rd to accomplish its mission. Indeed, Hageleuken et al. have observed “the docking of Rdx precisely at the tunneling hot spot of RdxR, and the absence of any appreciable conformational changes during Rdx binding”^76^.

Flexibility is frequently reported as key for the functioning of enzymes. As expressed by Tsou, “flexibility … is mandatory for the maximal expression of enzyme activity”^77^. Or, as articulated by Hammes and coworkers, flexibility is one of the “Pillars of Enzyme Catalysis”^78^. However, it is not clear how much flexibility is required for electron transfer proteins such as rubredoxin. Rubredoxin needs to dock with its respective electron donors and acceptors, but it may be that the electrostatic forces, perhaps combined with partner flexibility, are sufficient to bring the Rd Fe center close enough for electron transfer.

Evidence for Rd promiscuity can be seen in *Mycobacterium tuberculosis*, where the native rubredoxin (RubB) can shuttle electrons from two cognate reductases, FprA and FdR, to several different heme-based P-450 enzymes: CYP124, CYP125, and CYP142. Sushko et al*.* concluded that the rubredoxin interactions were “transient and not highly specific”^79^.

To reiterate, much of the discussion about the importance of appropriate flexibility of motion *vs*. temperature, relates to conformational changes needed to enable enzyme activity. However, we note that the rubredoxins that are the subject of the current study are electron transfer agents and conformational changes may not be critical for their activity. Thus, if the natural partners of the current rubredoxins merely require proximity of the Fe site to the donor or acceptor site, flexibility might not be a key determinant for its effectiveness. Perhaps we should consider rubredoxins as closer to reagents than to enzymes.

We note that the optimal growth conditions for organisms, their protein flexibility, and the thermal stability of their constituent proteins, while correlated, are mathematically related. Data for melting temperatures of psychrophile rubredoxins are scant, so instead we refer to the well-studied mesophile *Clostridium pasteurianum* rubredoxin – *Cp *Rd. Here the optimized growth temperature for *Cp* at pH 6.5 is 40 °C^80^, while *T*_m_ value for *Cp* Rd was reported as 104 °C^3^. This contrasts with the optimal growth temperature of 100 °C for *Pyrococcus furiosus*, while the *T*_m_ value for *Pf* Rd was reported as 104 °C^3^

Of course, flexibility might still be important for the range of temperatures over which Rd is stable, but that will involve thermodynamic issues instead of kinetic ones. Although the thermal stability of *Pg* Rd as not been investigated, it is known that mesophile *Cp* melts around 57 °C^81^, while *Pf* Rd melts at 144 °C^82^. NRVS at temperatures above 300 K will be difficult, and Mössbauer and NFS are out of the question. Furthermore, the studies have demonstrated that flexibility is variable across proteins^31^, and our current study has been limited to dynamics at the Fe site. We will need to employ other methods for answering such questions.

## Summary

We began this study in part as a test for the corresponding states hypothesis^83–85^. We found that psychrophilic rubredoxins and hyperthermophilic have comparable flexibility at the Fe site from 4 K to 300. It may be that flexibility is not essential to rubredoxin electron transfer, but additional studies at higher temperatures, perhaps by other techniques such as SRCD, SAXS, and NMR, are needed to examine the relationship between flexibility and thermal stability.

## Methods

### Production and purification of the recombinant rubredoxins

The recombinant *P. furiosus* rubredoxin was expressed and purified essentially as described^38^. Recombinant *P. glacialis* rubredoxin was purified in a similar way. Based on the amino acid sequence (WP_196868757.1) the gene was codon optimized for expression in *E. coli*, synthesized and cloned into plasmid pET24a by Genscript to generate plasmid pPglRd. The recombinant vector containing the gene that codes for a rubredoxin from *Pseudomonas* sp. AU10 (GenBank accession number OP536816) was synthesized with codon optimization for *E. coli* in GenScript. In order to substitute the natural abundance Fe with ^57^Fe, all proteins were expressed and purified as described above, with cultures that were supplemented with 50 μM ^57^Fe. Full experimental details are in the Supporting Information.

### NRVS measurements

NRVS spectra were measured and recorded using published procedures^86^ at either BL09XU, BL19LXU, or BL35XU. These beamlines employ two monochromators: first, a high heat load monochromator (HHLM) to produce ~ 1.0 eV resolution at the ^57^Fe nuclear resonance (14.41 keV) and second, a high-resolution monochromator (HRM) to reduce the bandwidth to 0.8 meV (6.5 cm^-1^)^87^. The photons from both the nuclear fluorescence and the internal conversion K shell fluorescence were recorded with a 2 × 2 APD array.

The ^57^Fe partial vibrational density of states (PVDOS), the real temperatures (T_r_), the Lamb-Mössbauer factors (LM) and other sum rule quantities^46^ for the samples were derived from the measured NRVS using the PHOENIX software package^88^. Full experimental details are in the Supporting Information.

### Mössbauer measurements

Mössbauer spectra were recorded by using a home-built spectrometer equipped with a Janis Research (Wilmington, MA) SuperVaritemp Dewar that allows measurements in the temperature range from 1.5 to 250 K and a constant-velocity transducer. The temperature was monitored by using a calibrated resistance type temperature sensor (Lake Shore Cernox CX-SD). The isomer shifts are quoted relative to α-Fe foil at 298 K. The velocity transducer was calibrated using a sodium nitroprusside standard.

### Nuclear forward scattering (NFS)

NFS measurements were performed at BL19LXU at SPring-8 using the same high-resolution monochromator and cryostat as NRVS measurements. Photons from the high-resolution monochromator were transmitted through the sample at about 1 m distance onto a 7-element APD detector array a further 1 m downstream. NFS spectra were measured using the SPring-8 D-mode time structure, comprising a single synchrotron pulse of 13 ps with an interval of 684.3 ns. The APD detector was capable of counting at 14 kHz (2 kHz per array element) and the incident photons were attenuated to optimize to this detector count rate using aluminum filters before the sample. Two kinds of measurement were taken, time spectra at a given temperature, and total count rate between ~ 18–600 ns as a function of temperature.

NFS spectra were simulated using custom spectrum fitting software. Lamb-Mössbauer factors were estimated from the both the simulated spectra and total count rates as described in the Supporting Information.

### Molecular dynamics calculations

Molecular dynamics simulations were carried out using GROMACS version 2021.5^89^^90^. Protein coordinates were obtained from a previous X-ray structural determination of the Rd from *P. furiosus* (PDB: 1BRF)^91^. The simulations utilized the CHARMM27^92^ forcefield for protein interactions. Van der Waals interactions for the cofactor were obtained from previous work on rubredoxin, using the nonbonding parameters for the bridging sulfurs and iron face^33^. The Fe-(Cys)4 interactions were adapted from empirical NRVS data^72^ that were previously simulated by VIBRATZ^93^. Charges for the Fe-(Cys)4 site were obtained from Density Functional Theory (DFT) calculations. The DFT geometry optimizations were performed with the program ORCA version 4.2^94^ using the BP86^95–97^ Customized with 10% exact Hartree–Fock exchange as functional and Ahlrich’s triple-zeta basis set Def2-TZVPP^98^ at the ORCA grid6 integration grid level and the CP(PPP)^99^ basis set for Fe atoms at the grid7 integration grid level. The DFT calculation used implicit solvation (CPCM) with ε = 4 and was corrected for dispersion effects (D3)^95^^,^^96^. Initial coordinates were taken from the XRD structures for Fe-(Cys)4 in 1BRF, then subsequently geometry optimized with the positions of all Cα carbons fixed to their starting positions^100^. The charges for the Fe-(Cys)4 moiety were then extracted by the CHarges from Electrostatic Potentials using a Grid (CHELPG) method^101^ implemented in ORCA. Individual charges were scaled to a summed net charge of -2, and integrated into the molecular dynamics forcefield. More complete details are in the Supporting Information.

### Supplementary Information

Supplementary Information.

## Data Availability

The data that support this study are available from the corresponding authors upon request.
