# Supplementary material for: Temperature-dependent iron motion in extremophile rubredoxins – no need for ‘corresponding states’
Source: Sci Rep. 2024 May 28;14:12197. doi: 10.1038/s41598-024-62261-2 (PMC11133467; doi:10.1038/s41598-024-62261-2)
Supplement: Supplementary file 1 — Supplementary Information. [file 41598_2024_62261_MOESM1_ESM.pdf]

SUPPORTING INFORMATION

Temperature-Dependent Iron Motion in Extremophile Rubredoxins – No Need for 'Corresponding States'

Francis E. Jenney Jr.,[†] Hongxin Wang,[$] Simon J. George,[$] Jin Xiong,[¶] Yisong Guo,[¶]
Leland Gee,[£] Juan José Marizcurrena,[‡] Susana Castro-Sowinski,[‡] Anna Staskiewicz,[†]
Yoshitaka Yoda,[¥] Kenji Tamasaku,[§] Nobumoto Nagasawa,[¥] Lei Li,[∞] Hiroaki Matsuura,[§]
Tzanko Doukov,[#] Michael Y. Hu,[¢] and Stephen P. Cramer[$,*]

[†] *Georgia Campus, Philadelphia College of Osteopathic Medicine, Suwanee, GA 30024*
[$] *SETI Institute, Mountain View, CA 94043*
[¶] *Department of Chemistry, Carnegie Mellon University, Pittsburgh, PA 15213*
[£] *LCLS, SLAC National Laboratory, Stanford, CA 94025*
[#] *SSRL, SLAC National Laboratory, Stanford, CA 94025*
[‡] *Universidad de la República, Montevideo, Uruguay*
[¢] *Advanced Photon Source, Argonne National Laboratory, Lemont, IL 60439*
[¥] *Precision Spectroscopy Division, SPring-8/JASRI, Sayo, Hyogo 679-5198, JAPAN*
[§] *RIKEN/SPring-8 Center, Hyogo 679-5148, Japan*
[∞] *Synchrotron Radiation Research Center, Hyogo 679-5165, Japan*

1. Methods

1.1 Hyperthermophilic *Pyrococcus furiosus (Pf)* Rubredoxin Sample Preparation

Construction and maintenance of the recombinant vector. The recombinant vector containing the gene that codes for a rubredoxin from *Pyrococcus furiosus* (Genbank accession number AAF03228.1) was synthesized with codon optimization for expression in *Escherichia coli* in plasmid pET24d by GenScript (Piscataway, NJ). The plasmid was electroporated into *E. coli* BL21(DE3) containing plasmid pSdef1, (a generous gift from Dr. David LeMaster) which overexpresses *E. coli* peptide deformylase [1] and maintained in LB medium with 50 ug/ml kanamycin, 34 ug/ml chloramphenicol.

Production and purification of the recombinant rubredoxin. The recombinant *P. furiosus* rubredoxin was expressed and purified essentially as described [2]. In order to substitute the natural abundance Fe with ^{57}Fe, purified rubredoxin was treated as described [3]. Protein was diluted to a concentration of 1-5 mg/ml and denatured in 20% w/v final concentration Trichloroacetic acid (TCA – added drop-wise while swirling) containing 0.5 M 2-mercaptoethanol (2-ME) and 50 mM EDTA. Once the red color has disappeared, and the protein had precipitated, the white precipitate was collected by centrifugation in oakridge tubes 50,000 x g 30min 4°C). The pellet was resuspended in the same solution with a glass rod and centrifuged. This was repeated 2x to remove any remaining Fe from the protein. The pellet was dried, then resuspended in ddH2O and centrifuged, 3-4 times to remove TCA.

The pellet was dissolved to 2-5 mg/ml in 500 mM Tris pH 8.0 with 140 mM 2-ME swirling 100 rpm 37°C.

The solubilized protein was transferred to an acid-washed serum bottle with a small stirrer, sealed, and the head space flushed with Ar. Metallic ^{57}Fe (Cambridge Isotope Laboratories, Cambridge, MA; 5x molar ratio:protein) was dissolved in 100 µl *aqua regia* (3:1 $HCl:HNO_3$), and once dissolved add 100 µl 1M NaOH. The ^{57}Fe solution was injected to the stirring protein (it turned dark brown immediately), and stirred under Ar at 25 °C overnight. The next morning, the sample was diluted to 50 ml in 100 mM Tris pH 8.0, and filtered through a 0.45 µm filter to remove precipitated Fe. The sample was then extensively washed to remove remaining Fe, and the buffer was exchanged to the desired solution using MilliporeSigma (Burlington, MA) Amicon Ultra 3 kDa MWCO concentrators at 3400 x *g* 15C. For NRVS experiments the protein was concentrated to ~10 mM. Protein concentrations were determined using the ε_{494nm} 9.22 $mM^{-1}cm^{-1}$ [4]. Reduced samples were generated by addition of a 5x molar excess of sodium dithionite (DT 50 mM) in 100 mM Tris pH 8.0 and washing extensively using Millipore Microcon 3 kDa MWCO in an anaerobic chamber. Reduced protein (~45 µl) was added to NRVS cuvettes with a syringe, sealed with a small amount of vacuum grease, removed from the chamber and flash frozen in liquid nitrogen. Oxidized (as purified) protein was loaded and frozen in NRVS cuvettes in the same way. Samples were shipped to the beamline in a dry liquid N_2 shipper.

1.2 Psychrophilic *Polaromonas glacialis (Pg)* Rubredoxin Sample Preparation

Construction and maintenance of the recombinant vector. Recombinant *Polaromonas glacialis* rubredoxin was expressed as described above for *Pf* rubredoxin. Based on the amino acid sequence (WP_196868757.1) the gene was codon optimized for expression in *E. coli*, synthesized and cloned into plasmid pET24a by Genscript (Piscataway, NJ) to generate plasmid pPglRd. The protein was overexpressed as described above for *Pf* rubredoxin. Cell pellets were resuspended in lysis buffer (50 mM Tris pH 8.0, 1 mM EDTA, 100 mM NaCl, 3 ml per gram wet weight of cell pellet) and frozen at -20°C. Cells were melted and sonicated on ice ~750 watts until liquid is free-flowing (~2 min per 10 ml of resuspended cells). Phenylmethylsulfonyl fluoride (PMSF) was added to a final concentration of 1 mM to this whole cell extract (WCE), and the WCE was centrifuged in 50 ml oakridge tubes 50,000 x *g* 30 minutes at 4°C.

Production and purification of the recombinant rubredoxin. Recombinant *Po. glacialis* rubredoxin was expressed as described above for *Pf* rubredoxin. Based on the amino acid sequence (WP_196868757.1) the gene was codon optimized for expression in *E. coli*, synthesized and cloned into plasmid pET24a by Genscript (Piscataway, NJ) to generate plasmid pPglRd. The protein was overexpressed as described above for *Pf* rubredoxin. Cell pellets were resuspended in lysis buffer (50 mM Tris pH 8.0, 1 mM EDTA, 100 mM NaCl, 3 ml per gram wet weight of cell pellet) and frozen at -20°C. Cells were melted and sonicated on ice ~750 watts until liquid is free-flowing (~2 min per 10 ml of resuspended cells). Phenylmethylsulfonyl fluoride (PMSF) was added to a final concentration of 1 mM to this whole cell extract (WCE), and the WCE was centrifuged in 50 ml oakridge tubes 50,000 x *g* 30 minutes at 4°C. The red supernatant was transferred to a bottle, ammonium sulfate was added to a final concentration of 25% w/v, and incubated on ice overnight. The next day, the extract was centrifuged in 50 ml oakridge tubes 50,000 x *g* 30 minutes at 4°C. The red supernatant was filtered through a 0.22 mM syringe filter, then, using an AKTA Start system, was loaded directly onto a Phenyl sepharoseHP column (Cytiva, Marlborough, MA)

equilibrated in buffer A (50 mM Tris pH 8.0 / 1.89 M $(NH_4)_2SO_4$). After loading, the column was washed with buffer A until the A_{280nm} returned to baseline. The protein was eluted with buffer B (50 mM Tris pH 8.0) using a gradient of 0-50% B over 2 column volumes (cv), followed by a gradient of 50-100% B over 5 cv. The red peak elutes between 650-0 mM $(NH_4)_2SO_4$). Red fractions were combined and concentrated in buffer B then loaded onto a HiPrep 26/60 Sephacryl S-100 column (Cytiva) equilibrated in 50 mM Tris pH 8.0 / 200 mM NaCl. The red peak was concentrated and buffer exchanged into 50 mM MOPS pH 7.0 using 15 ml Amicon 3 kDa MWCO concentrators (Fisher Scientific, Pittsburgh, PA). The protein was loaded onto a SourceQ15 column (Cytiva) equilibrated in 50 mM MOPS pH 7.0. After loading the column was washed with buffer A until the A_{280nm} returned to baseline. The red protein was eluted with buffer B (50 mM MOPS pH 7.0 / 1M NaCl) first using a gradient of 0-150 mM NaCl over 1 cv, then 150-500 mM NaCl in 9 cv. The protein elutes as two major peaks, peak 1 ~250-280 mM NaCl and peak 2 280-310 mM NaCl. Purity was confirmed by SDS-PAGE analysis. Recombinant rubredoxins typically are produced in multiple forms that differ in N-terminal processing in *E. coli* (Jenney & Adams 2001). Electrospray ionization (ESI) mass spectrometry indicated that peak 1 has a mass of 6008 Daltons (N-terminal methionine), and peak 2 has a mass of 6036 Da, consistent with an N-terminal formyl-methionine.

Isotopic labeling. For ^{57}Fe labeling, all proteins were expressed and purified as described above, except that the cultures were supplemented with 50 µM ^{57}Fe. ^{57}Fe (metal powder, Chemgas, Boulogne-Billancourt / France, 95.95% enriched)) was dissolved in *aqua regia* (3:1 HCl: HNO_3); once dissolved, an equal volume of 5N NaOH was added to neutralize the acid, and this was added directly to the culture flask just before growth.

1.3 Psychrophilic *Pseudomonas* sp. AU10 *(Px)* Rubredoxin Sample Preparation

Construction and maintenance of the recombinant vector. The recombinant vector containing the gene that codes for a rubredoxin from *Pseudomonas* sp. AU10 (GenBank accession number OP536816) was synthesized with codon optimization for *E. coli* in GenScript (https://www.genscript.com/; USA). We requested to include a C-terminal 6-His tag and a thrombin cleavage site and to fuse the construction into the expression vector pMAL-c5e(+), which encodes for an N-terminal MBP fusion.

For the maintenance of the recombinant vector, the construct was transformed to *Escherichia coli* DH5α chemo-competent cells [5]. For the recombinant production, the vector was transformed to *E. coli* BL21 Arctic Express (DE3) cells. Strains were stored in 20 % glycerol at −80 °C.

Production and purification of the recombinant rubredoxin. The recombinant *E. coli* BL21 Arctic Express (DE3) strain was grown in Luria Broth (10 g tryptone, 5 g yeast extract, 5 g NaCl) supplemented with glucose (0,4 % w/v) and ampicillin (100 mg mL^{-1}) at 37 °C, overnight (pre-culture). The expression was performed by inoculation of this pre-culture in 250 mL of the autoinduction Zym-5052 medium [6], at an initial turbidity of 0,4 at 600 nm. The medium was supplemented with ampicillin (100 mg mL^{-1}). First, cells grew at 37 °C for 4 h (to increase the biomass), and then the temperature was shift down to 14 °C for 48 h to allow protein expression. Cells were harvested by centrifugation (6000 g at 4 °C for 15 min) and suspended in 50 mM phosphate buffer, containing 300 mM NaCl and 10 mM imidazole, pH 8,2 (IMAC binding buffer). Cells were lysed by sonication (40 % amplitude, at a relative

power output of 10). The recombinant protein was purified from the soluble fraction (sf), obtained by centrifugation (16000 g at 4 °C, 30 min).

The AU10 recombinant rubredoxin (His-MBP-Px Rd) was purified by tandem affinity chromatography (immobilized metal affinity chromatography (IMAC) followed by an amylose affinity chromatography). For IMAC, the SF was loaded into a Co–NTA affinity resin (Invitrogen, Cat. No 89964) and the binding was done with a LKB 10200 Perspex Peristaltic Pump with a constant flux of 1.5 mL min^{-1} at RT. The resin was then washed with 5 column volumes of binding buffer and, the His-MBP-Px Rd protein was eluted using 1 column volume of binding buffer containing 150 mM imidazole. The eluate, containing the recombinant protein, was immediately loaded onto an amylose affinity matrix (NEB E8021S) the binding was done with a LKB 10200 Perspex Peristaltic Pump with a constant flux of 1.5 mL min^{-1} at RT. Then, the resin was washed with 50 mM phosphate buffer and the elution was performed with 1 column volume of 50 mM phosphate buffer supplemented with 10 mM maltose and 10 % glycerol.

The removal of the His-MBP fusion from the recombinant His-MBP-Px Rd protein (52 kDa) was achieved by the specific proteolytic cleavage with the Thrombin CleanCleave kit (Merck, Cat No RECOMT-1KT); the recombinant protein without the purification target fusion was named Px Rd (6 kDa). To pull apart the His-MBP (44,5 kDa) fusion, the product obtained after the specific proteolysis we used a 30 kDa cutoff Centricon (Merck, Cat. No UFC9030). The flowthrough containing Px Rd was concentrated using a 3 kDa cutoff Centricon (Merck, UFC9003). All the fractions obtained during the production and purification of Px Rd were controlled by SDS-PAGE [7]. Protein concentrations were determined by Bradford assay (Amresco, M172). The purified recombinant protein was stored in PBS buffer supplemented with 50 % glycerol at − 80 °C (flash-frozen with liquid nitrogen).

1.4 NRVS Measurements and Analysis

Measurements. NRVS spectra were measured and recorded using published procedures [8] [9] [10] [11] at nuclear resonant scattering beamlines at SPring-8 (BL09XU [12] or BL19LXU [12]). A high heat load monochromator (HHLM) was first used to produce radiation ~1.0 eV energy resolution at the 14.4 keV energy of the ^{57}Fe nuclear transition. A high energy resolution monochromator (HRM) [13] was then used to reduce the bandwidth from 1 eV down to ~0.8 meV.

During NRVS measurements, the samples were positioned inside a cryostat which was maintained at 15K or less using liquid helium (LHe). A heater inside the cryostat near the NRVS samples was used to set various nominal temperatures (T_s) between 15K and 360K. However, the real sample temperatures (T_r) [14] were derived from the anti-Stoke/Stoke ratios and often fell 10-30K higher than the nominal set temperatures. The vibrational energy regions to scan were: 1) -240 cm^{-1} to 560 cm^{-1} for the samples measured at T_s = 15 – 120 K; 2) -320 cm^{-1} to 750 cm^{-1} for the samples measured at T_s = 120 – 240 K; 3) - 400 cm^{-1} to 1000 cm^{-1} for the samples measured at T_s = 240 – 360 K. The selection is based on the fact that samples at higher temperatures can have more population at higher or more negative energy positions. As the incident radiation scans through the set energy region(s), the total intensities collected from both the nuclear fluorescence and the internally converted electron K shell fluorescence were recorded with a 2x2 APD array [8].

Analysis. Such collected raw counts *vs.* the vibrational energy ($E_{vib} = E - E_0$) form a raw NRVS spectrum that can be further processed to be converted to a partial vibrational density

of state (PVDOS) *via* PHOENIX software package or a web tool at spectra.tools (a variant form of using PHOENIX) [8] [15] [16]. During this conversion, the real sample temperature as well as other sum rule quantities, such Lambda-Mössbauer factors (LM) and average force constants can also be obtained [17]. With PHOENIX related analysis, the information is read from an associated output file: phoxout.log.

1.5 Mössbauer Spectroscopy

Mössbauer measurements employed custom sample holders. Spectra were recorded at CMU using a home-built spectrometer equipped with a Janis Research (Wilmington, MA) SuperVaritemp Dewar that allowed measurements in the temperature range from 1.5 to 250 K and a constant-velocity transducer. The temperature was monitored by using a calibrated resistance type temperature sensor (Lake Shore Cernox CX-SD). The isomer shifts are quoted relative to α-Fe foil at 298 K. The spectra were calibrated using a sodium nitroprusside standard.

1.6 Nuclear Forward Scattering

NFS Measurements. NFS measurements were performed at BL19LXU at SPring-8 using the same high-resolution monochromator and cryostat as NRVS measurements. Unlike NRVS measurements, NFS experiments have a standard transmission geometry. Photons from the high-resolution monochromator were transmitted through the sample at about 1 meter distance onto a 7-element APD detector array a further 1 meter downstream. NFS spectra were measured using the SPring-8 D-mode time structure, comprising a single synchrotron pulse of 13 ps with an interval of 684.3 ns. The high-resolution monochromator was tuned to the ^{57}Fe excitation energy (~14.41 keV) by measuring the NFS spectrum of a 10 μm Fe foil. The APD detector was capable of counting at 14 kHz (2 kHz per array element) and the incident photons were attenuated to optimize to this detector count rate using aluminum filters before the sample.

For these experiments, samples comprised rubredoxin with ~5 mM ^{57}Fe concentration mounted in small Delrin custom holders with pathlength ~2.7 mm, values that should give a good NFS spectra with relatively little distortion from dynamical beats. These sample holders were in turn were mounted in a custom copper cryostat sample holder. Since the sample and sample holder were mounted in a vacuum on the cryostat cold face, the sample holder was designed to ensure that the sample had both good thermal contact with thermostated copper on all sides as well as good thermal shielding along the X-ray path. The cryostat was cooled with either liquid nitrogen or liquid helium. After each temperature change, the total NFS count rate was monitored for each sample for 15–30 minutes to ensure the temperature had equilibrated across the entire sample.

NFS Analysis. Two kinds of NFS measurement were performed, a measure of the total count rate after a delay of approximately 18 ns, and time dependent spectra. Depending on the total count rate, a spectrum took between 20 minutes to 10 hours to measure. NFS spectra were analyzed using custom *Mathematica*™ and C scripts as discussed in the results section.

1.7 Molecular Dynamics Calculations

Molecular dynamics simulations were carried out using GROMACS version 2021.5 [18] [19]. Protein coordinates were obtained from a previous X-ray structural determination of the rubredoxin from *Pyrococcus furiosus* (PDB: 1BRF) [20]. The simulations utilized the

CHARMM27 [21] forcefield for protein interactions. Van der Waals interactions for the cofactor were obtained from previous work on rubredoxin, using the nonbonding parameters for the bridging sulfurs and iron face [22]. The Fe-$(Cys)_4$ interactions were adapted from empirical NRVS data [23] that were previously simulated by VIBRATZ [24]. Charges for the Fe-$(Cys)_4$ site were obtained from Density Functional Theory (DFT) calculations. The DFT geometry optimizations were performed with the program ORCA version 4.2 [25] using the BP86 [26] [27] [28] customized with 10% exact Hartree-Fock exchange as functional and Ahlrich's triple-zeta basis set Def2-TZVPP [29] at the ORCA grid6 integration grid level and the CP(PPP) [30] basis set for Fe atoms at the grid7 integration grid level. The DFT calculation used implicit solvation (CPCM) with $\varepsilon=4$ and was corrected for dispersion effects (D3) [26] [27]. Initial coordinates were taken from the XRD structures for Fe-$(Cys)_4$ in 1BRF, then subsequently geometry optimized with the positions of all Cα carbons fixed to their starting positions.[31] The charges for the Fe-$(Cys)_4$ moiety were then extracted by the Charges from Electrostatic Potentials using a Grid (CHELPG) method [32] implemented in ORCA. Individual charges were scaled to a summed net charge of -2, and integrated into the molecular dynamics forcefield.

The rubredoxin system was inserted into a rectangular box with a minimum distance of 20 Å between the protein and any boundary giving a 73 Å length for each cube dimension, solvated by water molecules (modeled by TIP4P-FB [33]), and neutralized with sodium and chloride ions to a concentration of 0.1M. The system was then energy minimized to <100kJ/mol. Next the simulation was distributed into 41 temperatures from 10K to 300K and at each temperature equilibration was achieved between the solvent and protein *via* isothermal-isochoric ensemble followed by isothermal-isobaric ensemble for 100 picoseconds each. Finally, the dynamics simulations were conducted at each temperature. Classical dynamics were then held at multiple temperatures using a velocity rescaling thermostat [34] with a time constant of 0.1 ps. The pressure of the system was held at 1 atm using a barostat with a time coupling constant of 0.5ps [35]. The total duration of each simulation was 10 ns with a time step of 1 femtosecond and saving trajectory snapshots every 500 femtoseconds.

Trajectories were analyzed using the python library MDAnalysis [36]. For every temperature, each frame of the 10 ns simulations were transformed to center the protein within the simulation box. Next, the protein in each of the trajectory frames was aligned to minimize the entire Fe-$(Cys)_4$ moiety RMSD of each frame relative to the reference frame arbitrarily chosen at 2 nanoseconds of simulation time. Finally, the aggregated Fe atom mean squared deviation (MSD) was determined from the time-averaged Fe atom position of these aligned simulation frames.

2. Sequence Comparison of *Pf* and *Pg* Rd.

```
Pf   MAKWVCKICGYIYDEDAGDPDNGISPGTKFEELPDDWVCPICGAPKSEFEKLED   54
Pg   -MTWMCLICGWIYDEALGSPEHGIAAGTPWSQVPMNWTCPECGARKEDFEMVQM   53
       .*:* ***:****  *.*::**: ** :.::* :*.** *** *.:** ::
```

Figure S1. Sequence comparison of the *Pf* and *Pg* Rds.

The sequences of the two rubredoxins are 81% similar, and while we hesitate to draw conclusions, there are some interesting differences. The *Pf* rubredoxin is more acidic, with

a predicted pI of 4.05 (https://web.expasy.org/compute_pi/), than the *Pg* protein with a predicted pI of 4.25. There are also apparent differences in overall hydrophobicity, (https://web.expasy.org/protscale/), although the number of aromatic residues is basically the same and in the same positions. Also of note, the *Pg* protein has 4 internal methionine residues, which are not found in the *Pf* protein.

We are hesitant to draw any firm conclusions from these observations without further comparison to other hyperthermophilic and psychrophilic proteins. For instance, the differences in hydrophobicity could be due to the need to bind and function with partner proteins.

4. Supporting Information References

[1.] "Increased peptide deformylase activity for N-formylmethionine processing of proteins overexpressed in *Escherichia coli*: application to homogeneous rubredoxin production", Tang, J. Z.; Hernandez, G.; LeMaster, D. M. *Prot. Expr. Pur.*, **2004**, *36*, 100-105.

[2.] "Rubredoxin from *Pyrococcus furiosus*", Jenney, F. E., Jr.; Adams, M. W. W. *Methods Enzymol.*, **2001**, *334*, 45-55.

[3.] "Spectroscopic studies of cobalt and nickel substituted rubredoxin and desulforedoxin", Moura, I.; Teixeira, M.; LeGall, J.; Moura, J. J. *J. inorg. Biochem.*, **1991**, *44*, 127-139.

[4.] "Determinants of protein hyperthermostability: purification and amino acid sequence of rubredoxin from the hyperthermophilic archaebacterium *Pyrococcus furiosus* and secondary structure of the zinc adduct by NMR", Blake, P. R.; Park, J. B.; Bryant, F. O.; Aono, S.; K., M. J.; Eccleston, E.; Howard, J. B.; Summers, M. F.; Adams, M. W. *Biochemistry.*, **1991**, *30*, 10885-10895.

[5.] "Purification of nucleic acids by extraction with phenol:chloroform", Sambrook, J.; Russell, D. W. *CSH protocols*, **2006**, *2006*.

[6.] "Protein production by auto-induction in high density shaking cultures", Studier, F. W. *Protein Expr. Purif.*, **2005**, *41*, 207-234.

[7.] "Cleavage of structural proteins during the assembly of the head of bacteriophage T4", Laemmli, U. K. *Nature*, **1970**, *227*, 680-685.

[8.] "Nuclear Resonance Vibrational Spectroscopy: A Modern Tool to Pinpoint Site-Specific Cooperative Processes", Wang, H. X.; Braun, A.; Cramer, S. P.; Gee, L. B.; Yoda, Y. *Crystals*, **2021**, *11*, 909 - 951.

[9.] "A strenuous experimental journey searching for spectroscopic evidence of a bridging nickel-iron-hydride in [NiFe] hydrogenase", Wang, H.; Yoda, Y.; Ogata, H.; Tanaka, Y.; Lubitz, W. *J. Syn. Rad.*, **2015**, *22*, 1334-1344.

[10.] "Machine learning concept in de-spiking process for nuclear resonant vibrational spectra – Automation using no external parameter", Wang, J.; Li, L.; Wang, H. **2022**, *119*, 103352.

[11.] "Tracking energy scale variations from scan to scan in nuclear resonant vibrational spectroscopy: In situ correction using zero-energy position drifts ΔEi rather than making in situ calibration measurements", Wang, J.; Yoda, Y.; Wang, H. **2022**, *93*, 095101.

[12.] "Upgrade of the nuclear resonant scattering beamline, BL09XU in SPring-8", Yoda, Y.; Imai, Y.; Kobayashi, H.; Goto, S.; Takeshita, K.; Seto, M. *Nucl. Inst. Meth. A*, **2012**, *206*, 83.

[13.] "High-resolution monochromator for iron nuclear resonance vibrational spectroscopy of biological samples", Yoda, Y.; Okada, K.; Wang, H.; Cramer, S. P.; Seto, M. *Jap. J. App. Phys.*, **2016**, *55*, 122401.

[14.] "Real sample temperature: a critical issue in the experiments of nuclear resonant vibrational spectroscopy on biological samples", Wang, H. X.; Yoda, Y.; Kamali, S.; Zhou, Z. H.; Cramer, S. P. *J. Synchrotron Rad.*, **2012**, *19*, 257-263.

[15.] "CONUSS and PHOENIX: evaluation of nuclear resonant scattering data", Sturhahn, W. *Hyp. Int.*, **2000**, *125*, 149-172.

[16.] "NRVS for Fe in Biology: Experiment and Basic Interpretation", Gee, L. B.; Wang, H. X.; Cramer, S. P., in *Fe-S Cluster Enzymes, Part B*; 1st ed.; David, S. S., Ed.; Academic Press: Cambridge, MA, 2018; Vol. 599, pp. 409-425.
[17.] "Moments in nuclear resonant inelastic x-ray scattering and their applications", Hu, M. Y.; Toellner, T. S.; Dauphas, N.; Alp, E. E.; Zhao, J. Y. *Phys. Rev. B*, **2013**, *87*, 064301.
[18.] "GROMACS: High performance molecular simulations through multi-level parallelism from laptops to supercomputers", Abraham, M. J.; Murtola, T.; Schulz, R.; Páll, S.; Smith, J. C.; Hess, B.; Lindahl, E. *SoftwareX*, **2015**, *1-2*, 19-25.
[19.] "GROMACS 4.5: a high-throughput and highly parallel open source molecular simulation toolkit", Pronk, S.; S., P.; Schulz, R.; Larsson, P.; Bjelkmar, P.; R., A.; Shirts, M. R.; Smith, J. C.; Kasson, P. M.; van der Spoel, D.; Hess, B.; Lindahl, E. *Bioinformatics*, **2013**, *29*, 845-854.
[20.] "Crystal structure of rubredoxin from *Pyrococcus furiosus* at 0.95 Å resolution, and the structures of N-terminal methionine and formylmethionine variants of *Pf* Rd. Contributions of N-terminal interactions to thermostability", Bau, R.; Rees, D. C.; Kurtz, D. M.; Scott, R. A.; Huang, H. S.; Adams, M. W. W.; Eidsness, M. K. *J. Biol. Inorg. Chem.*, **1998**, *3*, 484-493.
[21.] "CHARMM: The biomolecular simulation program", Brooks, B. R.; III, C. L. B.; Jr., A. D. M.; Nilsson, L.; Petrella, R. J.; Roux, B.; Won, Y.; Archontis, G.; Bartels, C.; Boresch, S.; Caflisch, A.; Caves, L.; Cui, Q.; Dinner, A. R.; Feig, M.; Fischer, S.; Gao, J.; Hodoscek, M.; Im, W.; Kuczera, K.; Lazaridis, T.; Ma, J.; Ovchinnikov, V.; Paci, E.; Pastor, R. W.; Post, C. B.; Pu, J. Z.; Schaefer, M.; Tidor, B.; Venable, R. M.; Woodcock, H. L.; Wu, X.; Yang, W.; York, D. M.; Karplus, M. *J. Comp. Chem.*, **2009**, *30*, 1545-1614.
[22.] "Molecular dynamics simulations of rubredoxin from *Clostridium pasteurianum*: Changes in structure and electrostatic potential during redox reactions", Yelle, R. B.; Park, N.-S.; Ichiye, T. *Proteins: Struc. Func. Gen.*, **1995**, *22*, 154-167.
[23.] "Normal Mode Analysis of *Pyrococcus furiosus* Rubredoxin *via* Nuclear Resonant Vibrational Spectroscopy (NRVS) and Resonance Raman Spectroscopy", Xiao, Y.; Wang, H.; George, S. J.; Smith, M. C.; Adams, M. W. W.; Francis E. Jenney, J.; Sturhahn, W.; Alp, E. E.; Zhao, J.; Yoda, Y.; Dey, A.; Solomon, E. I.; Cramer, S. P. *J. Am. Chem. Soc.*, **2005**, *127*, 14596-14606.
[24.] "Fully automated microcomputer calculation of vibrational spectra", Dowty, E. *Phys. Chem. Minerals*, **1987**, *14*, 67-79.
[25.] "The ORCA program system", Neese, F. *WIRES: Comp. Mol. Sci.*, **2012**, *2*, 73-78.
[26.] "A consistent and accurate *ab initio* parametrization of density functional dispersion correction (DFT-D) for the 94 elements H-Pu", Grimme, S.; Antony, J.; Ehrlich, S.; Krieg, H. *J. Chem. Phys.*, **2010**, *132*, 154104.
[27.] "Effect of the damping function in dispersion corrected density functional theory", Grimme, S.; Ehrlich, S.; Goerigk, L. *J. Comp. Chem.*, **2011**, *32*, 1456-1465.
[28.] "Density-functional exchange-energy approximation with correct asymptotic-behavior", Becke, A. D. *Phys. Rev. A*, **1988**, *38*, 3098-3100.
[29.] "Balanced basis sets of split valence, triple zeta valence and quadruple zeta valence quality for H to Rn: Design and assessment of accuracy", Weigend, F.; Ahlrichs, R. *Phys. Chem. Chem. Phys.*, **2005**, *7*, 3297-3305.
[30.] "Prediction and interpretation of the ^{57}Fe isomer shift in Mössbauer spectra by density functional theory", Neese, F. *Inorg. Chim. Acta*, **2002**, *337*, 181-192.
[31.] "Vibrational and electronic characterization of a diiron bridging hydride complex – a model for hydrogen catalysis", Gee, L. B.; Pelmenschikov, V.; Wang, H.; Mishra, N.; Liu, Y.-C.; Yoda, Y.; Tamasaku, K.; Chiang, M.-H.; Cramer, S. P. *Chem. Sci.*, **2020**, *11*, 5487-5493.
[32.] "Determining atom-centered monopoles from molecular electrostatic potentials. The need for high sampling density in formamide conformational analysis", Breneman, C. M.; Wiberg, K. B. *J. Comp. Chem.*, **1990**, *11*, 361-373.
[33.] "Building Force Fields: An Automatic, Systematic, and Reproducible Approach", Wang, L.-P.; Martinez, T. J.; Pande, V. S. *J. Phys. Chem. Lett.*, **2014**, *5*, 1885-1891.
[34.] "Canonical sampling through velocity rescaling", Bussi, G.; Donadio, D.; Parrinello, M. *J. Chem. Phys.*, **2007**, *126*, 014101.

[35.] "Molecular dynamics with coupling to an external bath", Berendsen, H. J. C.; Postma, J. P. M.; van Gunsteren, W. F.; DiNola, A.; Haak, J. R. *J. Chem. Phys.*, **1984**, *81*, 3684-3690.
[36.] "MDAnalysis: A toolkit for the analysis of molecular dynamics simulations", Michaud-Agrawal, N.; Denning, E. J.; Woolf, T. B.; Beckstein, O. *J. Comp. Chem.*, **2011**, *32*, 2319-2327.
